# Secretion properties, clearance, and therapy in airway disease

**DOI:** 10.1186/2213-0802-2-6

**Published:** 2014-03-10

**Authors:** Bruce K Rubin

**Affiliations:** Department of Pediatrics, Children’s Hospital of Richmond at VCU, Virginia Commonwealth University School of Medicine, 1001 East Marshall St., P.O. Box 980646, Richmond, VA 23298 USA

## Abstract

Chronic airway diseases like cystic fibrosis, chronic bronchitis, asthma, diffuse panbronchiolitis, and bronchiectasis are all associated with chronic inflammation. The airway mucosa responds to infection and inflammation in part by surface mucous (goblet) cell and submucosal gland hyperplasia and hypertrophy with mucus hypersecretion. Products of inflammation including neutrophil derived DNA and filamentous actin, effete cells, bacteria, and cell debris all contribute to mucus purulence and, when this is expectorated it is called sputum. Mucus is usually cleared by ciliary movement, and sputum is cleared by cough.

These airway diseases each are associated with the production of mucus and sputum with characteristic composition, polymer structure, and biophysical properties. These properties change with the progress of the disease making it possible to use sputum analysis to identify the potential cause and severity of airway diseases. This information has also been important for the development of effective mucoactive therapy to promote airway hygiene.

## Review

### Introduction

**Figure 1:**
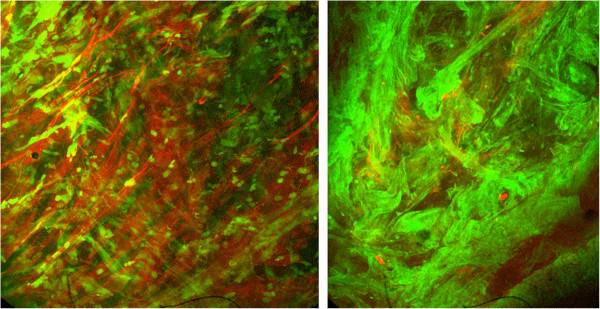
**Confocal micrograph showing mucin polymers (Texas red-UEA) and DNA polymers (Green - YoYo1) in bronchitis (left) and cystic fibrosis (right) sputum.**

Mucus clearance is a primary defense mechanism of the lung. Mucus is a barrier to airway water loss and microbial invasion and it is essential for the clearance of inhaled foreign matter [1]. Mucus is a viscoelastic gel consisting of water and high molecular weight glycoproteins, called mucins, mixed with serum and cellular proteins and lipids. The principal gel-forming mucins in the human airway are MUC5AC and MUC5B [2, 3]. There are variable amounts of cell debris and particulate matter in normal mucus. *Sputum* is expectorated mucus mixed with inflammatory cells, cellular debris, DNA and F-actin, as well as bacteria [4] (Figure 1). In cystic fibrosis (CF), there is almost no intact mucin in the airway secretions [5] due to mucin degradation by serine proteases [6]. These secretions are biochemically identical to pus.

Respiratory mucus is a mixture of submucous gland, goblet cell, and epithelial cell secretions [7]. Submucous glands are innervated by cholinergic, adrenergic, and non-adrenergic, non-cholinergic nerves [8]. Glandular secretions are thought to be the major constituent of respiratory mucus in health. Goblet cells expel their contents when exposed to irritants and appear to be inflammatory effector cells as well. Epithelial cells produce much of the periciliary fluid layer by active ion transport [9].

Mucus is usually cleared by airflow and ciliary interactions while sputum is primarily cleared by cough. Secretion clearance depends upon mucus properties such as viscoelasticity and adhesiveness, serous fluid properties, and ciliary function. In order to understand how impaired mucus clearance is related to respiratory disease and to develop new therapies, we study each of these properties as a function of disease type, disease severity, and therapy [10].

### Properties of mucus

#### Rheology

*Viscosity* is the loss of energy (loss modulus) from an object moving through a substance and thus the resistance to flow. This is a property of fluids and a Newtonian or ideal fluid can be rheologically defined by viscosity alone. *Elasticity* is the storage modulus and a property of solids. Pseudoplastic gels, like mucus gels are viscoelastic. Both viscosity and elasticity are essential for clearance [11]. While ciliary clearance can be impeded by high viscosity, cough clearance in increased when the gel is viscous and abhesive (not sticky) [12, 13].

#### Surface properties

Cohesivity is the ability of a substance to bind to itself or form threads under conditions of attachment deformation. Wettability is the surface energy at a solid-sputum-air interface and is measured using the sessile drop technique. Interfacial tension is the interfacial or surface energy between a gel and liquid or gas surface. This is also called surface tension when the interface is between an ideal liquid and gas [13].

Adhesivity is the ability to bond to a solid surface measured as the force of separation between one or more solid surfaces and the adhesive material. The work of adhesion is calculated from the contact angle and interfacial tension and tenacity or work of distraction is the product of cohesiveness and adhesiveness. Tenacity is the major physical determinant of cough transportability [14].

#### The transport properties of secretions

**Cough transportability** A simulated cough machine is used to measure the airflow-dependent clearability of sputum. Using this device, we have shown that cough clearability of secretions increases with greater mucus depth and the presence of periciliary fluid [15].

#### Ciliary function

Ciliary action is dependent on temperature, mucus gel and periciliary fluid hydration, mucus loading, and ciliary beat frequency, coordination, and power. Ciliary dysfunction can be due to congenital ciliary abnormalities such as primary ciliary dyskinesia [16], or caused by irritants, allergens, smoke, or infection. Mucociliary clearance is impaired both by mucus viscoelasticity and by mucus adhesiveness [17].

### Conditions associated with mucus clearance defects

#### Primary ciliary dyskinesia (PCD)

PCD leads to ineffective mucociliary clearance and bronchiectasis. These patients expectorate sputum with biophysical and clearance properties similar to sputum from persons with CF [18]. Similar to CF, there are high concentrations of inflammatory mediators in PCD sputum but a greater amount of intact mucin is present. It is thought that the much slower progression of disease in persons with PCD when compared with CF is probably due to conditions in the CF airway, which promote the growth of bacterial biofilms and chronic inflammation [19].

#### Cystic fibrosis (CF)

Sputum from persons with CF has *not* been shown to be abnormally thick, dehydrated, or viscous but has viscosity comparable to sputum from patients with bronchiectasis and less than that of persons with chronic bronchitis or severe asthma [20]. There are compelling data suggesting that surfactant abnormalities in CF sputum lead to secretions that are exceedingly adhesive [21]. Because of the CF abnormality in transepithelial chloride transport, it is likely that dysregulation of the depth and composition of the periciliary fluid layer may play a role in impeding mucus transport in the CF airway [22].

Data demonstrate that there is little intact mucin in the CF airway; even in young children with mild disease [5]. However the ability to secrete mucin appears to be intact and transiently expressed during periods of increased inflammation such as during an exacerbation of pulmonary infection [23].

#### Fucosidosis (congenital absence of alpha-L-fucosidase)

Fewer than 100 patients have been reported with this autosomal recessive defect due to absence of the gene for alpha-L-fucosidase on chromosome 1. Fucose and sialic acid are the predominant terminal sugars on mucin glycoprotein chains. We have described extremely watery mucus from the airway of a child with fucosidosis, and this mucus could not be easily cleared by cough or ciliary mechanisms [24]. The abnormal viscoelasticity is probably explained by defective mucus cross-linking.

#### Allergy and asthma

Inhalation of *Ascaris* antigen in low dose releases a large volume of watery mucus in sensitized dogs. When sufficient antigen is inhaled to cause bronchoconstriction, rigid, poorly cleared mucus is released [25]. This biphasic response is similar to that seen with the administration of cholinergic drugs and can be blocked with anticholinergics medications. A similar response can be seen in adults with acute asthma [26].

Mucus from patients with acute severe asthma has extremely high viscosity and patients who die during an asthmatic attack have extensive mucus plugging of their airways [27]. This is probably due to an abnormal secondary structure of the highly mucin enriched secretion [28, 29].

Patients with asthma who have chronic cough and sputum production have worse clinical control as measured by the asthma control questionnaire (ACQ) and more frequent exacerbations [26]. Helper T-cell type 2 (Th2) cytokines, including interleukin (IL)-13, are implicated in mucus production and goblet cell hyperplasia in asthma and IL-13 induces goblet cell hyperplasia with mucus hypersecretion in human airway epithelial cells. Airway goblet cell hyperplasia induced by IL-13 is steroid insensitive [30] but can be attenuated by 14- and 15- member macrolide antibiotics [31]. These data are consistent with clinical reports in steroid-resistant asthmatics [32]. Increased IL-13 mRNA expression is not reduced by steroid inhalation and under some circumstances; steroids further increase IL-13 induced mucin production [33].

Persons with severe asthma also have high levels of secretory phospholipases A2 (sPLA2) in their airway and bronchial lavage fluid. sPLA2 induce dramatic mucus hypersecretion, termed secretory hyperresponsiveness [34], cysteinyl leukotriene production, and hydrolyze airway surfactant [35].

#### Irritant exposure, smoking, bronchitis, and lung cancer

Acute exposure to irritants causes hypersecretion of watery, easily cleared mucus. Similarly, asymptomatic smokers produce watery mucus that is transported faster by cilia than normal mucus [36]. However *in vivo* mucociliary clearance is not increased in the light smoker probably because of epithelial damage.

Epidemiological studies show a correlation between COPD progression and chronic cough with sputum production. In The Copenhagen City Heart Study data analysis suggests that chronic mucus hypersecretion was significantly associated with both greater FEV1 decline and an increased risk of hospitalization [37]. It has been postulated that impaired mucus clearance in chronic smokers can lead to prolonged contact of irritants with the airway epithelium and so promote cellular metaplasia and cancer [38, 39].

#### Non CF bronchiectasis

The biophysical properties of sputum from children with bronchiectasis are different from those of subjects with CF or chronic bronchitis [40]. This leads to relatively greater cough transportability compared to CF sputum. These sputum properties may explain, in part, the different clinical course of children with idiopathic bronchiectasis compared to children with CF.

#### Plastic bronchitis

Plastic bronchitis is a rare disease in which there is the formation of large gelatinous or rigid branching airway casts. Plastic bronchitis has been associated with conditions as diverse as congenital heart disease almost always after palliative surgery, abnormalities of pulmonary lymphatic flow, sickle cell disease acute chest syndrome, and perhaps with hypersecretory severe asthma. The bronchial casts contain little or no DNA and consist almost entirely of fibrin and abnormally cross-linked mucin; similar to fatal asthma [41]. Because of the rarity of this disease, there are no reported randomized clinical trials of therapy although anecdotal case reports have suggested some benefit with the use of tPA acutely as a fibrinolytic agent, heparin aerosol chronically, presumably to inhibit Tissue Factor, and low dose macrolides to decrease excessive mucus production [42].

#### Therapy of mucus clearance disorders

The classification of mucoactive medications by presumed mechanisms of action is given in Table 1[43]. An overview of these medications and evidence of their effectiveness (or lack of effectiveness) is presented below.

**Table 1:** **Mucoactive medications and their presumed actions**

Mucoactive agent	Potential mechanisms of action
**Expectorants**	
Mannitol powder	
Hypertonic saline	Increases secretion volume and perhaps hydration
**Classical mucolytics**	
N-acetylcysteine	Severs disulfide bond linking mucin oligomers
Ambroxil	Increases chloride secretion and severs disulfide bonds
**Peptide mucolytics**	
Dornase alfa	Hydrolyzes DNA polymer with reduction in DNA length
Gelsolin or Thymosin β_4_	Depolymerizes F-actin
**Non-destructive mucolytics**	
Dextran	Breaks hydrogen bonds
**Mucoregulatory agents**	
Anticholinergic agents	Decreases volume of stimulated secretions
Glucocorticoids	Decreases airway inflammation and mucin secretion
Indomethacin	Decrease airway inflammation
Macrolide antibiotics	Decreases airway inflammation and mucin secretion
**Cough clearance promoters**	
Bronchodilators	Can improve cough by increasing expiratory flow
Surfactants	Decreases sputum adhesiveness

**Expectorants** are thought to increase the hydration of sputum either by the direct addition of water or by stimulation of water secretion into the airway [44]. Expectorants do not directly improve mucociliary clearance. The expectorants include water, guaifenesin, and the iodide containing medications. Despite widespread use of these agents, there are no well-controlled clinical trials that support their use, while randomized controlled trials have generally shown these medications to be ineffective [45–47].

Studies suggest that the inhalation of 7% saline can increase expectoration and improve pulmonary function in patients with CF [48]. This is probably due, in part, to stimulation of both water and mucus secretion into the hyperosmolar environment [49]. Mannitol, a sugar, has also been administered as a dry powder aerosol for the therapy of CF and non-CF bronchiectasis [50, 51]. Use of hypertonic saline is limited, in part, because of inflammation, cough, and airflow limitation (bronchospasm) in some patients [48]. Data suggest that hypertonic saline is less effective than dornase alfa (Pulmozyme) in improving pulmonary function in persons with CF [52].

Mucociliary clearance depends in part, on the viscoelasticity of the secretions. **Mucolytics** reduce sputum viscosity by disrupting polymer networks in the secretions. Classic mucolytic agents work by severing disulfide bonds, binding calcium, and depolymerizing mucopolysaccharides. Agents containing free sulfhydryl groups reduce the disulfide bridges interconnecting the mucin molecules. These agents include N-acetyl-L-cysteine (NAC) and similar drugs. There are no data that support the clinical use of aerosol NAC for the therapy of lung disease [53]. A large, prospective, randomized trial in subjects with chronic bronchitis showed no benefit of high dose oral NAC over placebo [54]. Orally administered NAC does not penetrate into the airway or bronchial lavage fluid [55] and inhaled NAC (pH 2.2) can cause bronchospasm and airway inflammation [56].

Sputum contains products of inflammation including DNA and filamentous actin (F-actin) polymers formed [4]. DNA and F-actin copolymerize to form a rigid network entangled with the mucin gel. Peptide mucolytics degrade these abnormal polymers. Dornase alfa (Pulmozyme, Genentech, South San Francisco, CA) is widely used for the treatment of CF airway disease and this peptide mucolytic has been shown to improve pulmonary function and decrease the frequency of pulmonary exacerbations when used daily as an aerosol [57, 58]. However dornase has not been shown to be effective in treating any pulmonary disease other than CF [59]. The G-actin sequestering and F-actin depolymerizing peptide, thymosin β4 has also shown promise *in vitro* as a peptide mucolytic and potentially an anti-inflammatory agent for the treatment of CF [60].

For cough to be effective there must be sufficient airflow to detach sputum from the epithelium and to mobilize secretions so that they can be expectorated. **Mucokinetic agents** improve the cough clearance of secretions, either by increasing airflow or by altering the sputum-epithelium interaction. By this definition, bronchodilators can be considered mucokinetic agents but *only* in patients who have a significant improvement in airflow with bronchodilator therapy.

Mucus adheres to the cilia and epithelium. It is thought that a bronchial surfactant layer promotes spreading of the mucous layer and efficient transfer of energy from beating cilia to the mucus preventing entanglement of the cilia in the mucus. Decreasing sputum tenacity increases sputum cough transportability in CF or chronic bronchitis. Some of the expectorant activity of classic mucolytics may be attributed un-sticking mucus from the airway surface.

Surfactant facilitates the spreading of the mucus across the tips of the cilia and it augments the efficient transfer of energy from the cilia to the mucous layer [13]. Surfactant therapy improves secretion transport in newborns with respiratory distress syndrome [61] and clinical trials with an aerosol surfactant in chronic bronchitis showed an improvement in pulmonary function and a decrease in trapped thoracic gas. This was associated with increased sputum mucociliary transportability [62].

**Mucoregulatory agents** inhibit mucus production or mucus secretion. Anticholinergic medications are the most well studied agents in this class. Anticholinergics can reduce the volume of *stimulated* secretions without increasing viscosity [63]. The topical anticholinergic bronchodilator, oxitropium bromide, has been shown to decrease the volume of secretions in patients with chronic bronchitis without changing mucus viscoelasticity [64]. The long-acting anticholinergic agent, tiotropium bromide, has kinetic selectivity for both the M1 and M3 receptor types over the M2 (auto-inhibitory) receptor [63]. In one study with COPD patients without exacerbations, tiotropium bromide treatment did not improve tracheobronchial clearance when compared with placebo [65].

Inflammation leads to mucous gland hyperplasia and many inflammatory mediators are potent secretagogues. Indomethacin has been administered by aerosol for the treatment of mucus hypersecretion in persons with diffuse panbronchiolitis, chronic bronchitis, or bronchiectasis [66]. Fourteen and 15-member macrolide antibiotics can reduce airway mucin secretion by virtue of their immunomodulatory activity [67–69]. This action is unrelated to the antibacterial activity and appears to be mediated by modulation of the ERK 1/2 pathway [70, 71]. Erythromycin and clarithromycin have been successfully used to treat mucus hypersecretion in patients with bronchorrhea, asthma, and sinusitis [72]. Low dose and long term azithromycin therapy is now routinely used for the therapy of CF [73].

#### The clinical use of mucoactive therapy

The principal indication for mucoactive therapy is to reduce airway obstruction by abnormal secretions. By decreasing the volume of secretions, gas trapping is reduced and there is improved performance of the muscles of respiration. Therapy should first be directed at decreasing infection and inflammation and minimizing exposure to irritants. The use of mucoactive medications and therapy to decrease mucus production and improve sputum expectoration can then be considered. Chest physical therapy, with or without accessory devices is usually prescribed along with the use of mucoactive medications [74].

Patients most likely to benefit from mucoactive therapy usually have a history of increased sputum expectoration and preserved airflow. Patients with acute mucus retention such as acute bronchitis or exacerbations of CF appear to be less responsive to mucoactive medications than stable patients. This may be due to decreased airflow caused both by the increase in infection and to muscular weakness in association with the pulmonary exacerbation, further reducing airflow dependent clearance mechanisms.

The effectiveness of therapy in an individual patient can be difficult to assess. When the patient feels better and there is an improvement in airflow or a reduction in trapped thoracic gas benefit is clear. However, changes in FEV1 poorly reflect clinical improvement with mucoactive therapy. Intuitively, one might expect that expectorated sputum volume would be a good way to assess the effectiveness of therapy but the expectorated sputum volume relates poorly, at best, to improvement in pulmonary function or the clinical status of the patient [75].

The scientific evaluation of secretion properties and response to therapy should enable the development of effective mucoactive therapy and allow us to better determine which patients are most likely to benefit from specific therapy.

## Conclusion

Many airway diseases are associated with the production of mucus and sputum with characteristic composition, polymer structure, and biophysical properties. These properties change with the progress of the disease making it possible to use sputum analysis to identify the potential cause and severity of airway diseases. This information has also been important for the development of effective mucoactive therapy to promote airway hygiene.
